# Oxidative Stress Biomarkers and Mitochondrial DNA Copy Number Associated with *APOE4* Allele and Cholinesterase Inhibitor Therapy in Patients with Alzheimer’s Disease

**DOI:** 10.3390/antiox10121971

**Published:** 2021-12-10

**Authors:** Chia-Wei Liou, Shih-Hsuan Chen, Tsu-Kung Lin, Meng-Han Tsai, Chiung-Chih Chang

**Affiliations:** 1 Department of Neurology, Kaohsiung Chang Gung Memorial Hospital and Chang Gung University College of Medicine, Kaohsiung 83301, Taiwan; cwliou@ms22.hinet.net (C.-W.L.); csho216@hotmail.com (S.-H.C.); tklin@adm.cgmh.org.tw (T.-K.L.); 2 Center for Mitochondrial Research and Medicine, Kaohsiung Chang Gung Memorial Hospital and Chang Gung University College of Medicine, Kaohsiung 83301, Taiwan; 3 Cognition and Aging Center and Institute for Translational Research in Biomedicine, Kaohsiung Chang Gung Memorial Hospital and Chang Gung University College of Medicine, Kaohsiung 83301, Taiwan

**Keywords:** Alzheimer’s disease, aolipoprotein genotype, TBARS, thiols, mtDNA copy number, mitochondrial haplogroup

## Abstract

Studies of the oxidative/anti-oxidative status in patients with Alzheimer’s disease (AD) carrying different alleles of the apolipoprotein E (*APOE*) gene are currently inconclusive; meanwhile, data regarding mitochondrial DNA copy number (mtCN) remain limited. We herein determined the thiobarbituric acid reactive substances (TBARS), thiols, and mtCN in blood samples of 600 AD patients and 601 controls. A significantly higher oxidative TBARS (1.64 μmol/L), lower antioxidative thiols (1.60 μmol/L), and lower mtCN (2.34 log Delta Ct) were found in the AD cohort as compared to the non-AD cohort (1.54 μmol/L, 1.71 μmol/L, 2.46 log Delta Ct). We further identified the ε4 alleles (*APOE4*) and separated subjects into three groups according to the number of *APOE4*. A significant trend was noted in the TBARS levels of both AD and non-AD cohorts, highest in the homozygous two alleles (1.86 and 1.80 μmol/L), followed by heterozygous one allele (1.70 and 1.74 μmol/L), and lowest in the no *APOE4* allele (1.56 and 1.48 μmol/L). Similar trends of lower thiols and mtCN were also found in the AD cohort. In our study of the influence of cholinesterase inhibitor therapy, we found significantly reduced TBARS levels, and elevated mtCN in AD patients receiving rivastigmine and galantamine therapy. Our study demonstrates associations between the *APOE4* allele and oxidative stress biomarkers and mtCN. Using cholinesterase inhibitor therapy may benefit AD patients through attenuation of oxidative stress and manipulation of the mtCN.

## 1. Introduction

Although many studies continue to investigate different etiologies of the various known forms of dementia, the cardinal pathological changes, primarily accumulations of amyloid beta and tau protein in the brain, have been demonstrated. These pathological changes, and their associations with oxidative stress, are common phenomena noted in neurodegenerative disease research and are promising targets for therapeutic intervention [1]. Previous studies have demonstrated marked elevations of oxidative stress markers in patients diagnosed with Alzheimer’s disease [2]. In addition, as with many diseases, genes play a fundamental role in the induction of Alzheimer’s disease. The identification of an association between individuals carrying the epsilon 4 allele of apolipoprotein E (*APOE4*) gene and an elevated risk of late-onset Alzheimer’s disease (AD) has been widely observed in population groups of various ethnicities [3]. While the underlying pathogenesis for this association remains undetermined, the induction of mitochondrial dysfunction and oxidative stress by the *APOE4* gene are factors which could be involved in a potential etiology [4].

Mitochondrial dysfunction and the resulting oxidative stress have been noted as significant factors leading to neuronal cell damage, and subsequent neurodegenerative disease [5]. Such dysfunction may be induced by mitochondrial DNA (mtDNA) variants, or by several other conditions influencing the functional expression of the mitochondrial organelle [6]. As previously reported, mutations in the mtDNA encoding the enzyme cytochrome c oxidase have been associated with AD [7]. Additionally, patients harboring some specific mitochondrial haplogroups, as defined by composites of specific mtDNA variants, have been reported to have an elevated risk for the generation of AD [8]. Meanwhile, several studies using cellular models, transgenic mouse models, and postmortem human brain tissue analyses have demonstrated associations between various mitochondrial functional expressions and risk of AD, specifically noted in carriers of the *APOE4* allele [9,10,11]. However, while the ultimate results of these dysfunctions are increased oxidative stress and subsequent cellular injury, study results regarding the oxidative status in patients carrying different *APOE* allele genotypes remain controversial [12,13,14]. The small case numbers, ethnic differences, environmental factors, and lifestyles may have contributed to the inconsistent results of past studies. Moreover, investigations into mtDNA copy number variations, which have been reported as a common response to oxidative stress, are also lacking with regard to *APOE* allele carriers.

The importance of oxidative stress in the pathology of AD is currently being investigated in relation to various etiologies. There is growing evidence indicating an interaction between gut microbiota and the brain through a number of neuro-chemical pathways which may interfere with short-chain fatty acids, 5HT, acetylcholine, tryptophan or ammonia [15,16,17]. Compelling evidence has suggested that any disturbance in these routes through the metabolites produced by gut microbiota and the associated modulation of the oxidative status of the central nervous system could potentially be associated with the development of AD [18,19].

In this study, we hypothesized that the differences of oxidative stress and mtDNA copy number levels found in patients carrying different allele genotypes of *APOE* could be applied as biomarkers to predict the onset of AD and to measure the effectiveness of therapeutic interventions. Herein, we report the results of our investigation on oxidative stress biomarkers and mtDNA copy number levels in AD patients carrying different *APOE* genotypes, as well as the influences of cholinesterase inhibitor therapy on these biomarkers.

## 2. Materials and Methods

### 2.1. Subjects

Two groups of Taiwanese subjects were enrolled in the study. Group 1 consisted of 600 patients (247 male and 353 female) with an average age of 76.4 ± 9.2 (standard deviation) years who had been diagnosed with AD. All patients were recruited from the outpatient clinics of the Cognitive and Aging Center at Kaohsiung Chang Gung Memorial Hospital. The diagnosis of AD was determined by the consensus of a multi-disciplinary team composed of behavioral neurologists, psychiatrists, neuropsychologists, neuroradiologists, and experts in nuclear medicine. The AD patients were diagnosed in accordance with the International Working Group-2 criteria [20]. Group 2 consisted of 601 age- and sex-matched control subjects (255 male and 346 female) with a mean age of 76.1 ± 8.4 years, who had participated in our health examination and had been examined clinically without significant signs of neurological or cognitive impairment related to AD. Written informed consent was obtained from all participants, in accordance with protocols approved by the institutional review board at the Kaohsiung Chang-Gung Memorial Hospital. The study was performed in accordance with the Declaration of Helsinki and its text revisions. Venous blood samples were collected after an overnight fasting. The DNA was isolated from leucocytes using PUREGENE^®^ DNA Purification kit (Gentra, MN, USA). The main characteristics of these patients and controls are summarized in Table 1. All AD patients were also checked for their usage of cholinesterase or non-cholinesterase inhibitor medication at the time of blood sample collection. The duration of use for three different kinds of cholinesterase inhibitor medication, including donepezil, rivastigmine, and galantamine or non-cholinesterase inhibitor, were then recorded. Patients were subsequently divided into subgroups based on their medication usage and medication type. Newly diagnosed AD patients with no history of medication were assigned into the non-medication group for comparison purposes.

### 2.2. Methods for Determination of Apolipoprotein E Genotype

The apolipoprotein E (*APOE*) gene polymorphism was determined by polymerase chain reaction-restriction fragment length polymorphism (PCR-RFLP) assay. In brief, 100ng of genomic DNA was amplified using the following primers (each 0.5 μL): 5′-TAAGCTTGGCACGGCTGTCCAAGGA-3′ and 5′-ACAGAATTCGCCCCGGCCTGGTACACTGCCA-3′. The polymerase chain reaction (PCR) was initiated with a 94 °C soak for 5 min followed by amplification for 30 cycles (94 °C for 30 s, 66.8 °C for 30 s and 72 °C for 40 s), and a final extension for 7 min at 72 °C using 5 μL Taq 5X Master Mix. The PCR product (10 μL) was digested at 1 μL HhaI for at least 2 h, the digested PCR product was resolved on an 8% nondenaturing acrylamide gel, which was electrophoresed at 105 V for 65 min. The gel was visualized under ultraviolet light to determine the *APOE* genotype [21]. All participants were categorized into three groups according to the number of E4 allele pattern of *APOE* gene. Group 1 contained no E4 allele; Group 2 contained heterozygous one E4 allele; and Group 3 contained homozygous two E4 alleles.

### 2.3. Methods for Determination of Mitochondrial DNA Haplogroup

The genomic DNA was extracted from blood leukocytes. Generally, 10 mL of venous blood was collected for the study. We used 24 pair primers to perform the gene amplification by multiplex PCR and 94 probes for mitochondrial haplogroup definition. Oligonucleotide probes were covalently bound to the carboxylated fluorescent microbeads using ethylene dichloride. Amplicons were labeled with SA-PE using the Eppendorf Mastercycler gradient after hybridization. Reactions were then measured by the Luminex100 flow cytometer [22]. We selected 40 mtSNPs which define 10 major haplogroups (A, B, C, D, E, F, G, M7, M8, N9) and their constitutive sub-haplogroups (B4, B5, D4, D5, F1, F2, M7a, M7b) in the Taiwanese population by referencing the human mtSNP database provided in the Mitomap website, and previously constructed phylogenetic trees for Chinese and Japanese populations [23,24]. The mtSNPs for the corresponding haplogroups have been illustrated in our previous report [25].

### 2.4. Assessment of Oxidative and Anti-Oxidative Stress Capacities

Serum free thiols were determined by directly reacting thiols with 5,5′-dithio-bis-(2-nitrobenzoic acid) (DTNB) to form 5-thio-2-nitrobenzoic acid (TNB). The thiols level was quantified from the absorbance using the extinction coefficient of TNB (A412 = 13,600 M^−1^ cm^−1^) [26]. The serum thiobarbituric acid reactive substance (TBARS) concentration was assessed following the method established by Ohkawa et al. [27]. The results are expressed as micromoles of TBARS per liter. A standard curve of TBARS was obtained by hydrolysis of 1,1,3,3-tetraethoxypropane (TEPP).

### 2.5. Measurement of Leukocyte mtDNA Copy Number

The mtDNA copy numbers were measured by real-time PCR, and were corrected by simultaneous measurement of the nuclear DNA. The forward and reverse primers for a nuclear gene, which are complementary to the β-actin gene, were 5′-TCACCCACACTGTGCCCATCTACGA-3′ and 5′-CAGCGGAACCGCTCATTGCCAATGG-3′. The forward and reverse primers for mtDNA, which are complementary to the sequence of the ND1 gene, were 5′-TGGGTACAATGAGGAGTAGG-3′ and 5′-GGAGTAATCCAGGTCGGT-3′. The PCR was conducted in an ABI PRISM 7700 Sequence Detection System (PE Biosystems, Waltham, CA, USA), using the SYBR^®^ GREEN PCR MASTER MIX kit (Applied Biosystems, Waltham, NJ, USA). The melting curves analysis was performed using the Dissociation Curve Software. In the same quantitative PCR run, the threshold cycle number (Ct) values of the β-actin gene and the mitochondrial ND1 gene were determined for each individual. Every measurement was performed at least three times, and normalized in each experiment against a serial dilution of a control DNA sample. In general, further measurements were required when samples did not meet the criteria of a standard deviation of less than 0.1. If no acceptable results were acquired, we gave up the sample and repeated with blood from another sample collection. In addition, patient data presenting unusually low or high levels were discarded. Ct values can be used as a measure of the input copy number and Ct value differences used to quantify mtDNA copy number relative to the ß-actin gene with the following equation: Relative copy number (Rc) = 2 (2 Ct), where Ct is the Ct ß-actin—Ct ND1 [28].

### 2.6. Statistical Analysis

Continuous variables are expressed as the mean ± standard deviation. Logarithmic transformation was applied to the data showing non-normal distribution. Group comparisons and trend were performed using the Student’s *t*-test, Pearson’s linear regression and one-way ANOVA. *p* < 0.05 was considered statistically significant. As mtDNA copy number displayed a non-linear distribution pattern, we changed it to a delta CT set for comparison; whereas oxidative stress markers displayed in a linear distribution. The contrast factor was applied in a one-way analysis of variances to test for linear trends displayed by the number of *APOE4* allele number subgroups. Statistical analysis was performed using the Statistical Package for Social Science program (SPSS for Windows, version 11.5; SPSS, Chicago, IL, USA).

## 3. Results

### 3.1. Differences between Demographics, Comorbidities, and Biological Markers of AD and Non-AD Cohorts

More subjects with a history of hypertension and a higher BMI were identified in the non-AD group than the AD group. No significant differences between the two groups were found regarding age, sex, or other medical conditions. Importantly, a significantly higher average level of serum oxidant TBARS (1.62 ± 0.73 μmol/L vs. 1.54 ± 0.86 μmol/L, *p* = 0.003), and a lower average level of serum anti-oxidant thiols (1.64 ± 0.46 μmol/L vs. 1.71 ± 0.39 μmol/L, *p* < 0.001) were noted in the AD cohort as compared with the non-AD controls. The average mtDNA copy number was also noted to be significantly lower in the AD cohort than the non-AD controls (2.34 ± 0.21 vs. 2.46 ± 0.28, *p* < 0.001). The differences between these biomarkers remained consistent after adjustment for age, sex, BMI, smoking, and other medical conditions (see Table 1).

### 3.2. Differences between Levels of Biological Markers of Various APOE Allele Groups in the AD and Non-AD Cohort

**Figure 1:**
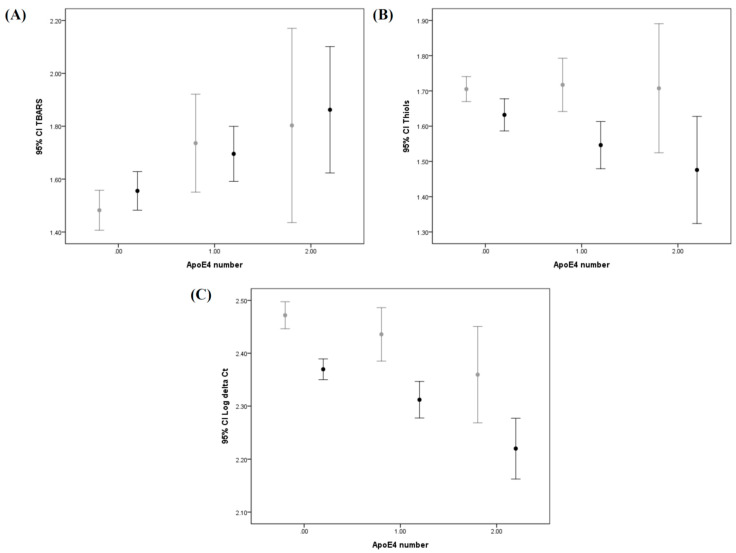
Differences between levels of biological markers of various *APOE4* allele groups in the AD and non-AD cohorts: Error bar plots show levels of average TBARS (**A**), thiols (**B**), and log delta Ct copy number (**C**) in different *APOE4* allele subgroups of AD patients and non-AD controls. ● in **A**–**C** represents AD patient group; ● in **A**–**C** represents non-AD control group).

Distributions of the *APOE* allele pattern in the AD and non-AD cohorts are shown in Table 2. A significant association between the presence of the E4 allele and AD was noted as more prominent in the homozygous allele than the heterozygous allele. Further investigations into the oxidative marker revealed levels of TBARS were 1.56 ± 0.72, 1.70 ± 0.73, 1.86 ± 0.71 in the three individual *APOE4* groups of the AD cohort, and 1.48 ± 0.84, 1.74 ± 0.94, 1.80 ± 0.79 in the individual *APOE4* groups of the non-AD cohort (Table 2). More specifically, a trend was noted wherein higher TBARS levels were directly correlated to the number of E4 alleles (Figure 1A, Ptrend = 0.003 and Ptrend = 0.003 in AD and non-AD, respectively). Studies for the anti-oxidative marker showed levels of thiols were 1.63 ± 0.45, 1.55 ± 0.47, 1.48 ± 0.45 in the three individual *APOE4* groups of the AD cohort, and 1.71 ± 0.40, 1.71 ±0.38, 1.71 ± 0.39 in the individual *APOE4* groups of the non-AD cohort. These differences reached statistical significance only in the AD cohort (Figure 1B, Ptrend = 0.008). Meanwhile, our study of the mtDNA copy number in the peripheral leukocytes found 2.37 ± 0.19, 2.31 ± 0.24, 2.22 ± 0.17 in the three individual *APOE4* groups of the AD cohort, and 2.47 ± 0.29, 2.44 ± 0.26, 2.36 ± 0.20 in the non-AD cohort. A significant trend of lower mtDNA copy number was correlated with presence of the *APOE4* allele in the AD cohort (Figure 1C, Ptrend < 0.001); however, this trend did not reach statistical significance in the non-AD cohort (Figure 1C, Ptrend = 0.053).

### 3.3. Association Study for the Relationship between Specific mtDNA Haplogroups and AD as Well as Oxidative Stress and mtDNA Copy Number

Among the twelve coding region variant-determined mtDNA haplogroups and eight of their sub-haplogroups in the Taiwanese population, we found only the M7 and F2 haplogroups to reveal borderline associations with generation of AD. However, these associations were rendered insignificant after the Bonferroni correction (Table 3). This correction was used to correct for multiple comparisons of mtDNA haplogroups. Since we examined 16 haplogroups (A, B4, B5, C, D4, D5, E, F1, F2, G, M7b, M7c, M8, N9, others in N, others in M), we divided 0.05 by 15 to arrive at 0.0031. Thus, a p-value of < 0.0031 was considered statistically significant. Further, we studied the differences of average TBARS, thiols, and mtDNA copy number between the various mitochondrial haplogroups. We found no significant differences among both the AD and non-AD cohorts after comparing the individual haplogroups with the largest F haplogroup.

### 3.4. Investigation into the Effects of Cholinesterase Inhibitors on Oxidative Stress and mtDNA Copy Number

Changes of various oxidative stress biomarkers and mtDNA copy number in AD patients receiving cholinesterase inhibitors were studied. We identified 416 patients having received cholinesterase inhibitors for more than 3 months, including donepezil (144 patients), rivastigmine (176 patients), and galantamine (96 patients). Meanwhile, 141 newly diagnosed AD patients with no history of medication were assigned into the non-medication control group. A total of 43 cases having received non-cholinesterase inhibitor therapy, mainly memantine, or cholinesterase inhibitor therapy for less than 3 months, or those patients switching between different kinds of cholinesterase inhibitors within the 3 months prior to the study were categorized as the Others group. We then compared levels of oxidative/anti-oxidative biomarkers and mtDNA copy number between the various groups. A significantly lower TBARS level and higher mtDNA copy number were found in patients receiving rivastigmine and galantamine therapy as compared to the non-medication group. A higher mtDNA copy number was also found in patients receiving donepezil therapy as compared to the non-medication group (Table 4). However, differences of thiols levels were not significant between all three medication groups and the non-medication group. In addition, we found no differences between the three *APOE4* allele genotypes in terms of a lower TBARS level as well as a higher mtDNA copy number in patients receiving cholinesterase inhibitors (see Supplementary Table S1 and Figure 1).

## 4. Discussion

This large cohort case-control study provides evidence of an association between *APOE4* and oxidative stress which could play a significant role in the generation of AD. In addition to the finding of a higher average serum oxidative TBARS level in AD patients, we demonstrated a higher serum TBARS level in the AD-susceptible carriers of the *APOE4* genotype. The higher TBARS level was found in *APOE4* carriers of both the AD and non-AD cohorts, while it was more prominent in subjects carrying the homozygous allele. These data are consistent with previous findings from human group studies, and support previous observations of the increased oxidative status in human brain tissue samples of *APOE4* carriers, as well as in transgenic mice and cellular model studies [13,14,29,30]. The association of *APOE4* carriers with higher oxidative TBARS level noted here has previously been linked to the discrepant pleiotropic anti-oxidative functional expression of different *APOE* alleles [4,31]. Indeed, *APOE* has been identified to affect amyloid-β (Aβ) degradation, possibly via a complex system of increased Aβ trafficking to lysosomes [32]. However, this function is less effective with the *APOE4* genotype, leading to amyloid beta deposition, thereby inducing oxidative stress and consequent neurotoxicity, a major pathology of AD. In this study, we also demonstrated a lower average serum anti-oxidant thiols level in AD patients. A significantly lower serum thiols level was also found in AD patients carrying *APOE4*. Serum thiols is known as a primary free radical scavenger, which participates in the maintenance of the redox homeostasis within cells and plays important roles in various pathophysiological processes; furthermore, it is often applied as a biomarker to assess latent anti-oxidant capacity [33]. Our present study indicates serum thiols can be a simple and accurate method to measure anti-oxidative stress capacity, as compared to current methods.

In our study of mitochondrial copy number, we identified a significantly lower mitochondrial copy number in the blood samples of AD patients as compared to those in the non-AD cohort. A lower mitochondrial copy number has previously been reported in the peripheral leukocytes, cerebrospinal fluid, and post-mortem brain tissue samples of AD patients [34,35,36]. As mitochondrial DNA is the template for replication, and its variants may affect expressions of copy number, we therefore conducted association studies to clarify the role of mtDNA coding region variant-determined haplogroups in the changes of mtDNA copy number and possible correlation with AD. Although the M7 and F2 haplogroups were found to have borderline associations with generation of AD, the significance was lost after the multiple group comparison. In a further study of individual mtDNA haplogroups found within the Taiwanese population, we identified no associations between the generation of AD or differences of average mtDNA copy number. Thus, different average mtDNA copy numbers between the AD and non-AD cohorts cannot be attributed to mtDNA variant-determined haplogroup. Interestingly, a significant association between the number of *APOE4* alleles and mtDNA copy number was identified. As the *APOE4* allele has been indicated as a significant risk factor for generation of AD, it is reasonable to suggest the lower mtDNA copy number in the AD cohort of our study could be due to the presence of the *APOE4* allele. Indeed, translocase of outer mitochondrial membrane 40 (*TOMM40*), which lies in linkage disequilibrium with the *APOE* gene, has previously been reported to be associated with Alzheimer’s disease [37]. A recent study reported an association between elevated *TOMM40* RNA levels and decreased mitochondrial copy number as well as mitochondrial membrane potential in oxidative stress-challenged cells [38]. However, clarification of the mechanisms underlying the interaction between *APOE4* and *TOMM40* awaits further study.

During the past two decades, pharmacological therapy primarily using anticholinesterase inhibitors has become the standard treatment strategy for AD patients due to the various beneficial effects, including improved cognitive function and delayed disease progression [39]. Administration of these medications can increase the levels of the deficient neurotransmitter, acetylcholine, thereby extending and sustaining the impulse transmissions between neuronal synapses [40]. Recent reports have also found that while these medications are primarily applied for cholinesterase inhibition, they also affect multimodal actions [41]. In our study into the effects of medication on the oxidative/anti-oxidative status and mtDNA copy number, we found significant associations between AD patients using rivastigmine or galantamine and a lower serum TBARS level as well as increased mtDNA copy number. The reduction of TBARS level induced by cholinesterase inhibitors has previously been observed in both mouse and human studies; furthermore, it has been suggested that the reduction may inhibit disease progression [42,43]. Our study is the first to involve dementia patient observations to support this theory. In addition to reduced TBARS, we also observed elevated mtDNA copy numbers in patients receiving all three cholinesterase inhibitors, indicating the possible triggering of mitochondrial biogenesis. Indeed, previous studies have demonstrated that donepezil can increase the hepatic expression of PGC-1α and enhance mitochondrial biogenesis via AMP-activated protein kinase [44]. Increased mtDNA copy number and its associated compensation of an insufficient energy supply has been suggested as a potential therapeutic modality in the management of neurodegenerative diseases, including AD [45]. Therefore, the present findings may offer valuable insight into the potential role of mitochondrial biogenesis for the development of future AD therapies.

Gut dysbiosis has been proposed as an etiology in patients with AD [46,47]. Indeed, several gut microbiota have been reported to be associated with cognitive functions and neuropsychiatric symptoms in patients with AD [48]. However, the composition and abundance of gut microbiota have distinct presentations between countries and cultures. For example, data from American AD patients have demonstrated elevated measurements of Bacteroidetes and lower measurements of Actinobacteria in the phylum level [46], while in Chinese AD groups, lower measurements of Bacteroidetes and elevated measurements of Actinobacteria have been noted [49]. These inconsistencies create challenges to investigations of the associations between AD and gut microbiota; however, gut microbiota and the related modifications to oxidative stress in the etiology of AD offer a promising direction for future research and therapeutic strategies. It is worth noting that our study did not investigate the interactions between changes of gut microbiota and mitochondrial copy number; in addition, there is currently limited research into the potential associations between gut microbiota and the oxidative stress biomarkers we investigate here. It is possible that causal relationships between oxidative stress and mitochondrial copy number or drug responses may be influenced by gut microbiota. The correlations between microbiota bioactivity and bioavailability of pharmacological compounds may help to further clarify responses to cholinesterase inhibitors in future investigations.

## 5. Conclusions

Our study identified correlations between the presence of *APOE4* alleles and serum levels of oxidative TBARS and antioxidative thiols. In addition, we found a correlation between *APOE4* and mtDNA copy number in leukocytes. These biomarkers are affected in patients receiving cholinesterase inhibitor therapy. Thus, our study indicates that detection of *APOE4* and measurement of these biomarkers may be valuable tools for the monitoring of therapeutic responses in patients with AD in clinical practice.

## Figures and Tables

**Table 1:** Demographics, thiobarbituric acid reactive substances, thiols, mitochondrial DNA copy number, and apolipoprotein E4 allele of Alzheimer’s disease patients and controls.

Variable	AD Patients	Non-AD Controls	*p*
	(*n* = 600)	(*n* = 601)	
Age, year (SD)	76.4 (9.2)	75.6 (8.4)	0.087
Male, number (%)	247 (41.2)	260 (43.3)	0.462
BMI, mean (SD)	23.98 (3.57)	24.93 (3.79)	<0.001 *
Medical history			
Hypertension (%)	310 (51.7)	405 (67.4)	<0.001 *
Diabetes mellitus (%)	143 (23.8)	158 (26.3)	0.326
Smoking (%)	96 (16.0)	105 (17.5)	0.495
TBARS, μmol/L (SD)	1.64 (0.73)	1.54 (0.86)	0.003 *
Thiols, μmol/L (SD)	1.60 (0.46)	1.71 (0.39)	<0.001 *
mtDNA copy number, log Delta Ct (SD)	2.34 (0.21)	2.46 (0.28)	<0.001 *
*APOE4* allele			
Carrier of ε4: E2E4, E3E4, E4E4 (%)	224 (37.3)	111 (18.5)	
Non-carrier of ε4: E2E2, E2E3, E3eE3 (%)	376 (62.7)	480 (79.9)	<0.001 *

* Significance at the 95% confidence level (*p* < 0.05). Abbreviations: SD, standard deviation; BARS, thiobarbituric acid reactive substances; mtDNA, mitochondrial DNA; *APOE4*, apolipoprotein E4.

**Table 2:** Comparison of thiobarbituric acid reactive substances, thiols, mitochondrial DNA copy number between AD and non-AD cohorts carrying different numbers of apolipoprotein E4 alleles.

Variable	Number	TBARS	Thiols	mtDNA Copy Number
	(%)	μmol/L (SD)	μmol/L (SD)	Log Delt Ct (SD)
Alzheimer’s Disease (AD)				
No E4 allele (ε2ε2, ε2ε3, ε3ε3)	376 (62.7)	1.56 (0.72)	1.63 (0.45)	2.37 (0.19)
One E4 allele (ε2ε4, ε3ε4)	188 (31.3)	1.70 (0.73)	1.55 (0.47)	2.31 (0.24)
Two E4 allele (ε4ε4)	36 (6.0)	1.86 (0.71)	1.48 (0.45)	2.22 (0.17)
Non-Alzheimer’s Disease (non-AD)				
No E4 allele (ε2ε2, ε2ε3, ε3ε3)	480 (79.9)	1.48 (0.84)	1.71 (0.40)	2.47 (0.29)
One E4 allele (ε2ε4, ε3ε4)	101 (16.8)	1.74 (0.94)	1.71 (0.38)	2.44 (0.26)
Two E4 allele (ε4ε4)	20 (3.3)	1.80 (0.79)	1.71 (0.39)	2.36 (0.20)

Abbreviations: SD, standard deviation; TBARS, thiobarbituric acid reactive substances; mtDNA, mitochondrial DNA.

**Table 3:** Multivariate logistic regression analysis of mitochondrial haplogroups associated with Alzheimer’s disease (with adjustment for age and sex).

Haplogroup	AD (*n* = 600)	Non-AD (*n* = 601)	Total (*n* = 1201)	Multivariate	
	% (*n*)	% (*n*)	% (*n*)	Odds Ratio (95% CI)	*p*
Major haplogroup					
A	4.5 (27)	4.5 (27)	4.5 (54)	0.90 (0.51–1.58)	0.719
B	18.8 (113)	21.5 (129)	20.1 (242)	0.84 (0.63–1.13)	0.246
C	1.8 (11)	1.5 (9)	1.7 (20)	0.97 (0.39–2.42)	0.952
D	18.5 (111)	18.5 (111)	18.5 (222)	1.04 (0.77–1.40)	0.824
E	1.2 (7)	1.8 (11)	1.5 (18)	0.69 (0.26–4.81)	0.444
F	19.7 (118)	18.8 (113)	19.2 (231)	1.06 (0.79–1.43)	0.693
G	2.5 (15)	2.8 (17)	2.7 (32)	0.92 (0.44–1.92)	0.826
M7	16.0 (96)	11.3 (68)	13.7 (164)	1.43 (1.01–2.02)	0.042
M8	4.2 (25)	6.5 (39)	5.3 (64)	0.64 (0.38–1.09)	0.099
N9	2.7 (16)	4.3 (26)	3.5 (42)	0.61 (0.32–1.16)	0.133
Others N	2.2 (13)	1.7 (10)	1.9 (23)	1.44 (0.61–3.39)	0.41
Others M	8.0 (48)	6.8 (41)	7.4 (89)	1.24 (0.79–1.95)	0.346
Sub-haplogroup					
B4	12.5 (75)	14.3 (86)	13.5 (162)	0.89 (0.63–1.25)	0.497
B5	4.7 (28)	6.2 (37)	5.4 (65)	0.78 (0.46–1.30)	0.337
D4	11.5 (69)	11.2 (67)	11.3 (136)	1.03 (0.71–1.48)	0.896
D5	6.7 (40)	6.7 (40)	6.7 (80)	1.16 (0.72–1.85)	0.543
F1	10.0 (60)	9.0 (54)	9.5 (114)	1.13 (0.76–1.68)	0.557
F2	5.2 (31)	3.5 (21)	4.3 (52)	1.78 (1.02–3.11)	0.042
M7b	8.0 (48)	5.0 (30)	6.5 (78)	1.54 (0.95–2.49)	0.081
M7c	5.8 (35)	3.3 (20)	4.6 (55)	1.57 (0.89–2.76)	0.121

**Table 4:** Effects of cholinesterase inhibitor therapy on thiobarbituric acid reactive substances, thiols, and mitochondrial DNA copy number of Alzheimer’s disease patients.

Variable	Number	TBARS	Thiols	mtDNA Copy Number
	(%)	μmol/L (SD)	μmol/L (SD)	Log Delt Ct (SD)
Medications groups				
Donepezil	139 (23)	1.64 (0.65)	1.57 (0.46)	2.35 (0.20) *
Rivastigmine	169 (28)	1.52 (0.70) *	1.63 (0.44)	2.35 (0.21) *
Galantamine	94 (16)	1.49 (0.69) *	1.58 (0.44)	2.39 (0.21) *
Others	57 (10)	1.91 (0.98)	1.63 (0.46)	2.32 (0.22)
Non-medication group	141 (24)	1.68 (0.70)	1.58 (0.48)	2.30 (0.22)

* Significance at the 95% confidence level in comparison with the non-medication group (*p* < 0.05). Abbreviations: SD, standard deviation; TBARS, thiobarbituric acid reactive substances; mtDNA, mitochondrial DNA.

## Data Availability

The data presented in this study are available in this manuscript.

## Supplementary Materials

The following are available online at https://www.mdpi.com/article/10.3390/antiox10121971/s1, Table S1. Effects of anti-cholinesterase inhibitor therapy on thiobarbituric acid reactive substances, thiols, mitochondrial DNA copy number of Alzheimer disease patients stratified by different allele type of APOE4.
