# Supplementary material for: Oxidative Stress Biomarkers and Mitochondrial DNA Copy Number Associated with APOE4 Allele and Cholinesterase Inhibitor Therapy in Patients with Alzheimer’s Disease
Source: Antioxidants (Basel). 2021 Dec 10;10(12):1971. doi: 10.3390/antiox10121971 (PMC8750673; doi:10.3390/antiox10121971)
Supplement: Supplementary file 1 [file antioxidants-10-01971-s001.zip › antioxidants-1465419-supplementary.pdf]

Supplementary Table S1. Effects of anti-cholinesterase inhibitor therapy on thiobarbituric acid reactive substances, thiols, mitochondrial DNA copy number of Alzheimer disease patients stratified by different allele type of APOE4.

Medication group<br>Variant	Case number<br>(%)	Non-Medication<br>Average (case no)	Donepezil<br>Average (case no)	Rivastigmine<br>Average (case no)	Galantamine<br>Average (case no)	Others<br>Average (case no)	Overall<br>Average (case no)
mtDNA							
No E4 allele (ε2ε2, ε2ε3, ε3ε3)	376 (62.7)	2.35 ± 0.22 (93)	2.38 ± 0.18 (78)	2.35 ± 0.18 (114)	2.41 ± 0.18 (62)	2.42 ± 0.20 (29)	2.37 ± 0.19 (376)
One E4 allele (ε2ε4, ε3ε4)	188 (31.3)	2.20 ± 0.19 (38)	2.32 ± 0.24 (53)	2.39 ± 0.25 (46)	2.39 ± 0.24 (28)	2.24 ± 0.22 (23)	2.31 ± 0.24 (188)
Two E4 allele (ε4ε4)	36 (6.0)	2.20 ± 0.20 (10)	2.30 ± 0.17 (8)	2.23 ± 0.16 (9)	2.16 ± 0.20 (4)	2.17 ± 0.11 (5)	2.22 ± 0.17 (36)
TBARS							
No E4 allele (ε2ε2, ε2ε3, ε3ε3)	376 (62.7)	1.61 ± 0.66 (93)	1.62 ± 0.69 (78)	1.42 ± 0.67 (114)	1.45 ± 0.67 (62)	1.96 ± 1.08 (29)	1.56 ± 0.72 (376)
One E4 allele (ε2ε4, ε3ε4)	188 (31.3)	1.82 ± 0.78 (38)	1.69 ± 0.60 (53)	1.66 ± 0.74 (46)	1.60 ± 0.75 (28)	1.68 ± 0.85 (23)	1.70 ± 0.73 (188)
Two E4 allele (ε4ε4)	36 (6.0)	1.86 ± 0.62 (10)	1.54 ± 0.62 (8)	2.00 ± 0.66 (9)	1.23 ± 0.48 (4)	2.65 ± 0.59 (5)	1.86 ± 0.71 (36)
Thiols							
No E4 allele (ε2ε2, ε2ε3, ε3ε3)	376 (62.7)	1.61 ± 0.46 (93)	1.61 ± 0.51 (78)	1.69 ± 0.42 (114)	1.57 ± 0.41 (62)	1.64 ± 0.46 (29)	1.63 ± 0.45 (376)
One E4 allele (ε2ε4, ε3ε4)	188 (31.3)	1.49 ± 0.56 (38)	1.49 ± 0.40 (53)	1.54 ± 0.47 (46)	1.61 ± 0.47 (28)	1.70 ± 0.41 (23)	1.55 ± 0.47 (188)
Two E4 allele (ε4ε4)	36 (6.0)	1.56 ± 0.40 (10)	1.67 ± 0.42 (8)	1.33 ± 0.30 (9)	1.47 ± 0.72 (4)	1.28 ± 0.59 (5)	1.48 ± 0.45 (36)
